# Association between geriatric nutritional risk index (GNRI) and asthma in elderly individuals aged 60 and above: a cross-sectional study of the NHANES 2005–2018

**DOI:** 10.1186/s12890-025-03830-7

**Published:** 2025-09-24

**Authors:** Jue Wang, ZiMeng Wang, Qi Zhang, Shiting Yu

**Affiliations:** 1 The First Affiliated Hospital of Heilongjiang University of Chinese Medicine, Harbin, 150040 China; 2 Heilongjiang Red Cross SenGong General Hospital, Harbin, 150040 China

**Keywords:** Geriatric nutritional risk index (GNRI), Asthma, Cohort analysis, NHANES, XGBoost machine learning, Over 60 years old

## Abstract

**Objective:**

The geriatric nutritional risk index (GNRI) is a promising tool for predicting nutrition-related complications in older adults. This study aimed to explore the association between GNRI and asthma in individuals aged 60 and above.

**Methods:**

A retrospective cohort study was conducted using the National Health and Nutrition Examination Survey (NHANES) database. Propensity score matching was used to manage observational data to minimize clinical data bias and confounding variables. Weighted logistic regression with subgroup and sensitivity analyses was used to analyze the potential relationship between GNRI and asthma in elderly individuals aged 60 and above.

**Results:**

The study population consisted of individuals aged 60 and above. After adjusting for race, education, emphysema, and chronic bronchitis, the odds ratio (OR) for asthma in relation to the GNRI was 1.021 (95% confidence interval [CI]: 1.016–1.026, *P* < 0.001), indicating that a lower GNRI is associated with a higher risk of asthma in elderly individuals.The GNRI quartile analysis revealed a significant upward trend (Q4 versus Q1, OR: 1.666, 95% CI: 1.41–1.972, *P* < 0.001). The significance of the selected factors was assessed using the XGBoost machine learning model, which demonstrated that the GNRI was one of the top five variables influencing the risk of asthma in elderly individuals. Subgroup analysis confirmed the association between GNRI and factors such as gender, race, smoking, alcohol consumption, education level, poverty income ratio, emphysema, and chronic bronchitis. Furthermore, GNRI levels were associated with increased eosinophils, basophils, white blood cells, red blood cells, neutrophils, monocytes, and albumin levels.

**Conclusion:**

This study demonstrates that GNRI levels are significantly associated with asthma in the elderly.

**Supplementary Information:**

The online version contains supplementary material available at 10.1186/s12890-025-03830-7.

## Introduction

Asthma, a common chronic respiratory disease, progressively affects the elderly population [1]. The onset of asthma is influenced by numerous factors, and previous research has demonstrated that genetic factors and long-term exposure to environmental pollutants may act as risk factors for asthma. Nutritional status may also be a significant risk factor for asthma exacerbation in elderly individuals; however, relevant reports are currently lacking [2]. Studies have indicated that fetal malnutrition significantly increases the risk of developing asthma in adulthood and is associated with a decline in lung function, emphasizing the long-term impact of early nutritional deficiency on respiratory health in later life [3]. Malnutrition is a common health problem among older adults, and prolonged undernutrition can result in immune dysfunction and increase susceptibility to various diseases [4]. Low protein intake and deficiencies in vitamins and trace elements (such as vitamin D, zinc, and magnesium) may exacerbate airway inflammation and oxidative stress, promoting asthma development. Chen et al. simulated a malnutrition state using a protein-restricted diet to construct a mouse model of early-life malnutrition [5]. The findings revealed that these mice exhibited increased susceptibility to experimental asthma in adulthood, with stronger airway inflammation and higher reactivity. Further investigation revealed that CD4^+^T lymphocytes exhibited an increased glycolysis rate upon activation in early-life malnourished mice. Blocking the glycolysis pathway significantly suppressed the function and differentiation of these cells, and experimental asthma symptoms were alleviated. Some studies suggest that malnutrition is associated with a decline in lung function and worsening symptoms in patients with asthma. Min et al. reported that body mass index (BMI) and asthma prevalence were related to gender and that both malnutrition and obesity contributed to the pathogenesis of asthma [6]. Niu et al*.* reviewed randomized controlled trials on the effects of vitamin D on human asthma and concluded that vitamin D supplementation could reduce the frequency of acute asthma exacerbations [7]. Moreover, Guo et al. demonstrated that vitamin D alleviates airway inflammation in asthmatic mice by regulating the Th17/Treg balance via inhibiting the NF-κB pathway using an ovalbumin-sensitized asthma mouse model and vitamin D treatment [8]. However, it has been observed that numerous nutrients may be associated with the onset of asthma, but previous studies have primarily focused on single nutritional factors. A new comprehensive indicator that considers multiple nutritional aspects is required to facilitate a more holistic assessment of the relationship between nutrition and asthma and to assess the potential risk of asthma development.

Despite existing research suggesting a potential link between malnutrition and asthma, some limitations remain. First, the methods for assessing nutrition are limited, as BMI alone does not reflect the underlying malnutrition or sarcopenia. Second, the heterogeneity of the research samples was insufficient, with factors such as age and gender not being fully considered. Finally, there is a lack of long-term effect assessments, as most studies have focused only on short-term outcomes, resulting in insufficient evidence for interventions in patients with chronic asthma. Moreover, nutritional status is a critical factor in exploring management strategies for asthma in elderly patients, in addition to traditional pharmacological treatments and disease control. Therefore, optimizing the nutritional status of the elderly population has an undeniable impact on improving asthma symptoms and their quality of life.

To address these shortcomings, this study aimed to explore the relationship between the Geriatric Nutritional Risk Index (GNRI) and asthma in individuals aged 60 and above and to analyze its potential inflammatory mechanisms. GNRI is a nutritional assessment index based on weight, height, and serum albumin (Alb) levels and has been widely used to assess the nutritional status of older adults [9]. Previous studies have demonstrated that a low GNRI is closely associated with the occurrence of various chronic diseases in the elderly, such as cardiovascular diseases and diabetes [10, 11]. However, there remains a lack of systematic research on the relationship between GNRI and asthma in the elderly, particularly regarding its specific association with inflammatory responses. Studies have indicated that malnutrition is closely associated with increased inflammation, which is one of the core pathological mechanisms of asthma [12, 13]. Consequently, this study aimed to investigate the predictive value of the GNRI for asthma and its correlation with inflammatory biomarkers to provide new clinical evidence for the prevention and management of asthma in the elderly.

There is currently limited research on the relationship between GNRI and asthma in the elderly, particularly regarding the specific link between GNRI and asthma-related inflammatory markers. This study aimed to explore the relationship between GNRI, asthma, and inflammation levels in elderly individuals to evaluate the effect of nutritional status on the pathogenesis of asthma in this population. By analyzing the clinical characteristics and inflammatory markers of elderly patients with asthma with different GNRI levels, this study aimed to provide scientific evidence for nutritional intervention strategies for elderly patients with asthma.

## Materials and methods

### Data and preprocessing

The the National Health and Nutrition Examination Survey (NHANES) is an ongoing, cross-sectional, nationally representative survey conducted by the National Center for Health Statistics (NCHS) with the approval and sponsorship of the Center for Disease Control and Prevention. The survey collects data every two years using a complex multi-stage probability sampling procedure. Data were collected through face-to-face interviews with participants and physical examinations at Mobile Examination Centers, where blood and urine samples were also collected. The NHANES was approved by the NCHS Research Ethics Review Board, and all participants provided informed consent. This study used data from seven NHANES cycles (2005–2018), including asthma diagnosis data for participants.

**Fig. 1:**
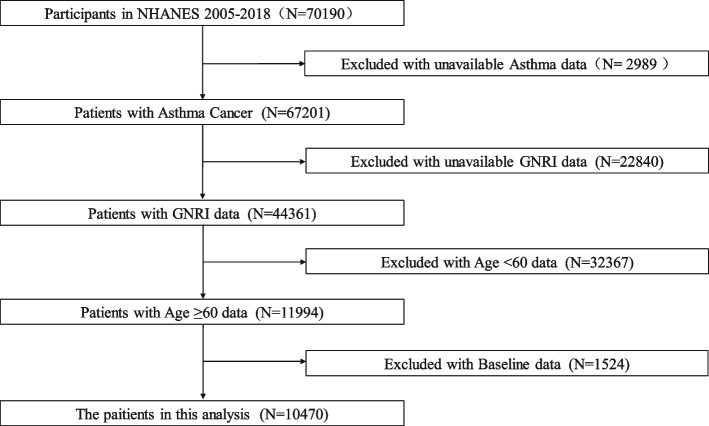
Flow diagram

From the initial 70,190 participants, the following exclusions were made: those without asthma diagnosis data (*N* = 2989), those without GNRI data (*N* = 22,840), those aged under 60 years (*N* = 32,367), and those lacking baseline data (*N* = 1524). The final study cohort included 10,470 participants, with 1,352 patients with asthma and 9,118 controls (Fig. 1).

### Definition of asthma in individuals aged 60 and above

#### Asthma definition

Asthma was diagnosed based on self-reported medical history. The Medical Condition Questionnaire (MCQ010) specifically inquired,"Have you ever been told by a doctor that you have asthma?"to identify patients with asthma. Non-asthmatic individuals were defined as those who answered negatively [14].

Furthermore, the relationship between the GNRI and inflammatory markers, such as eosinophil count (Eo count), basophil count (Ba count), white blood cell count (WBC count), red blood cell count (RBC count), neutrophil count (Neutrophil count), Alb, globulin (Globulin), and monocyte count (Monocyte count), was analyzed to explore potential mechanisms linking GNRI to asthma in elderly individuals.

#### Definition of elderly individuals aged 60 and above

Older adults were defined as those aged 60 and above, as determined from the demographic file (DEMO) in the NHANES dataset [15].

### Geriatric nutritional risk index (GNRI)

The GNRI, a simple nutritional index, was first introduced in 2005 and is closely associated with the prognosis of diseases such as diabetes, heart failure, and cancer [16, 17]. Previous studies have demonstrated that the GNRI significantly correlates with bone mineral density and osteoporosis. In the Chinese population, GNRI values increase with higher bone mineral density levels [18].

The GNRI formula is as follows: GNRI = (1.489) × serum Alb (g/L) + 41.7 × [weight/ideal weight], where ideal weight = 22 × height (m) × height (m). If the actual weight exceeds the ideal weight, the weight/ideal weight is set to 1. GNRI nutritional risk levels: High Nutritional Risk (GNRI < 98); Low Nutritional Risk (GNRI ≥ 98).

### Covariates

Potential confounding factors were identified based on previous literature, including age, gender, race, education level, poverty-income ratio, smoking, alcohol use, height, weight, BMI, emphysema, and chronic bronchitis.

Race, education level, and poverty income ratio were obtained from the DEMO in the NHANES database. Ethnic groups were categorized as Mexican–American, non-Hispanic Black, non-Hispanic White, other Hispanic, and other/multiple races. Education level was divided into five categories: < 9th Grade, 9th–11th Grade, high school, some college, and college graduate or higher. Household income was categorized as low-income (< 4.99) and high-income (≥ 5) based on the poverty income ratio.

### Weighted logistic regression analysis

Weighted binary logistic regression was used to analyze the potential association between GNRI and asthma in individuals aged 60 and above. GNRI was included in the regression model as both continuous and categorical variables. The continuous GNRI was divided into quartiles and treated as a categorical variable for further analysis. The lowest quartile (Q1) was used as the reference group. Three statistical models were established.

Model 1: Unadjusted model; Model 2: Adjusted for race and education level; Model 3: Further adjusted for emphysema and chronic bronchitis diagnoses. All regressions, including survey weights and non-normally distributed continuous covariates, were transformed using weighted quartiles for analysis.

### Restricted cubic splines (RCS)

RCS was used to model the relationship between continuous variables and outcomes, allowing for nonlinear associations while maintaining smoothness and avoiding overfitting.

### Clinical subgroup analysis

Subgroup analysis was performed to evaluate whether the association between GNRI and asthma varied across different subgroups (gender, race, smoking, alcohol consumption, education level, poverty income ratio, emphysema, and chronic bronchitis). Forest plots were used to visualize and compare the effect sizes and confidence intervals (CIs) across multiple subgroups.

### Sensitivity analysis

Sensitivity analysis was performed to assess the robustness of the association between the GNRI and asthma, ensuring the reliability of the findings.

### Statistical analysis

All data processing and analyses were performed using R software (version 4.1.1). Non-normally distributed continuous variables are presented as medians and interquartile ranges, and categorical variables are reported as sample counts and weighted percentages. The Wilcoxon rank-sum test was used to compare GNRI quartiles for continuous variables, and the Rao-Scott chi-square test was used for categorical variables. XGBoost machine learning was used to evaluate the importance of various factors in predicting the risk of asthma. Statistical significance was set at *P* < 0.05.

## Results

### Baseline characteristics of the sample

The study included 10,470 participants, of whom 1,352 were elderly individuals (aged 60 and above) diagnosed with asthma, and 9,118 were in the control group. The two groups exhibited significant differences in their baseline characteristics. The mean age of the asthma group was significantly lower than that of the control group (69 ± 7 versus 70 ± 7 years, *P* < 0.001). In terms of racial distribution, the proportion of non-Hispanic white individuals in the asthma group (49%) was significantly lower than that in the control group (51%, *P* < 0.001). Regarding gender distribution, the asthma group exhibited a significantly lower proportion of males (42%) than the control group (51%, *P* < 0.001).

Regarding height, the asthma group exhibited significantly shorter stature than the control group (164 ± 10 versus 165 ± 10 cm, *P* = 0.001). Considering weight, the asthma group exhibited a significantly higher average weight (83 ± 22 kg versus 79 ± 19 kg, *P* < 0.001). Similarly, the asthma group exhibited a significantly higher BMI than the control group (30.9 ± 7.7 versus 28.9 ± 5.9, *P* < 0.001).

The prevalence of emphysema (13% versus 3.1%, *P* < 0.001) and chronic bronchitis (28% versus 4.7%, *P* < 0.001) was significantly higher in the asthma group than in the control group. The GNRI, a novel nutritional indicator, was significantly higher in the asthma group (120 ± 14 versus 117 ± 11, *P* < 0.001), implying that a higher GNRI may be associated with an increased risk of asthma in elderly individuals aged 60 and above (Table 1).

**Table 1:** Baseline characteristics of asthma in NHANES (2005–2018)

Variable	Overall	Asthma	Non-asthma	*P*-value^*2*^
Age (years)	70 (7)	69 (7)	70 (7)	< 0.001
Gender				< 0.001
Male	5,253 (50%)	564 (42%)	4,689 (51%)	
Female	5,217 (50%)	788 (58%)	4,429 (49%)	
Race				< 0.001
Hispanic American	1,284 (12%)	129 (9.5%)	1,155 (13%)	
Other Hispanic	933 (8.9%)	136 (10%)	797 (8.7%)	
Non-Hispanic White	5,345 (51%)	659 (49%)	4,686 (51%)	
Non-Hispanic Black	2,097 (20%)	325 (24%)	1,772 (19%)	
Other races	811 (7.7%)	103 (7.6%)	708 (7.8%)	
Educational level				0.071
Less Than 9th Grade	1,669 (16%)	202 (15%)	1,467 (16%)	
9th–11th Grade	1,503 (14%)	200 (15%)	1,303 (14%)	
High School	2,520 (24%)	305 (23%)	2,215 (24%)	
AA Degree	2,663 (25%)	384 (28%)	2,279 (25%)	
College Graduate	2,115 (20%)	261 (19%)	1,854 (20%)	
PIR				0.31
Low	8,720 (83%)	1,139 (84%)	7,581 (83%)	
High	1,750 (17%)	213 (16%)	1,537 (17%)	
Smoke				0.67
Smoke	1,348 (13%)	179 (13%)	1,169 (13%)	
No Smoke	9,122 (87%)	1,173 (87%)	7,949 (87%)	
Drink				0.23
Drink	5,339 (51%)	669 (49%)	4,670 (51%)	
No Drink	5,131 (49%)	683 (51%)	4,448 (49%)	
Height (cm)	165 (10)	164 (10)	165 (10)	0.001
Weight (kg)	80 (19)	83 (22)	79 (19)	< 0.001
BMI (kg/m^2^)	29.1 (6.2)	30.9 (7.7)	28.9 (5.9)	< 0.001
Emphysema				< 0.001
Emphysema	457 (4.4%)	174 (13%)	283 (3.1%)	
Health	10,013 (96%)	1,178 (87%)	8,835 (97%)	
Chronic bronchitis				< 0.001
Chronic bronchitis	804 (7.7%)	372 (28%)	432 (4.7%)	
Health	9,666 (92%)	980 (72%)	8,686 (95%)	
GNRI	117 (12)	120 (14)	117 (11)	< 0.001

*BMI* Body mass index, *GNRI* Geriatric nutritional risk index

^a^Mean (SD) or Frequency (%)

^b^Wilcoxon rank sum test; Pearson's chi-squared test

### Relationship between asthma and GNRI in elderly individuals aged 60 years or older

A multivariable logistic regression model determined the association between asthma and GNRI in individuals aged 60 and above (Table 2). In Model 1 (unadjusted), a significant positive association was observed between the GNRI and asthma in the elderly (odds ratio (OR) = 1.021, 95% confidence interval [CI]: 1.017–1.026, *P* < 0.001). When the GNRI was categorized by quartiles, individuals in the third quartile (Q3, GNRI: 116.06–123.43) and fourth quartile (Q4, GNRI ≥ 123.43) exhibited a significantly higher risk of asthma than those in the first quartile (Q1, GNRI < 109.35), with ORs of 0.974 (95% CI: 0.821–1.156, *P* = 0.7636) and 1.658 (95% CI: 1.418–1.941, *P* < 0.001), respectively. Trend analysis revealed a significant positive correlation between increasing GNRI and asthma in the elderly (*P* for trend < 0.001). Although Model 2 was adjusted for race and education level, it still demonstrated a significant association between the GNRI and asthma (OR = 1.022, 95% CI: 1.017–1.026, *P* < 0.001). Compared with Q1, the risk of asthma in individuals in Q3 and Q4 continued to increase, with OR of 0.988 (95% CI: 0.831–1.174, *P* = 0.8922) and 1.674 (95% CI: 1.429–1.964, *P* < 0.001), respectively. The trend analysis results further supported the positive association between GNRI and asthma in the elderly (*P* for trend < 0.001). In Model 3, after adjusting for emphysema and chronic bronchitis, the association between GNRI and asthma remained significant (OR = 1.021, 95% CI: 1.016–1.026, *P* < 0.001). Subgroup analysis revealed that Q3 (GNRI: 116.06–123.43) exhibited an increased risk of asthma compared with Q1, but this association was statistically non-significant (OR = 1.067, 95% CI: 0.89–1.278, *P* = 0.4827). However, Q4 remained statistically significant (OR = 1.666, 95% CI: 1.41–1.972, *P* < 0.001). Trend analysis indicated that the increasing GNRI remained positively associated with asthma in the elderly, even after adjusting for covariates (*P* for trend < 0.001).

**Table 2:** The relationship between asthma and GNRI (multifactor regression OR (95% CI))

Variable	Model 1	Model 2	Model 3
GNRI	1.021 (1.017, 1.026)	1.022 (1.017, 1.026)	1.021 (1.016, 1.026)
*P*-value	< 0.001	< 0.001	< 0.001
GNRI, quartile			
Q1 (< 109.35)	1 (Ref)	1 (Ref)	1 (Ref)
Q2 (109.35–116.06)	1.004 (0.847, 1.191)	1.013 (0.854,1.201)	1.064 (0.889, 1.273)
Q3 (116.06–123.43)	0.974 (0.821, 1.156)	0.988 (0.831,1.174)	1.067 (0.89, 1.278)
Q4 (≥ 123.43)	1.658 (1.418, 1.941)	1.674 (1.429,1.964)	1.666 (1.41, 1.972)
*P*-trend	< 0.001	< 0.001	< 0.001

Model 1 was the crude model

Model 2 was adjusted for race and education

Model 3 was adjusted for race, education, chronic bronchitis, heart rate, and emphysema

*OR* Odds ratio, *CI* Confidence interval, *GNRI* Geriatric nutritional risk index

### Machine learning analysis of factors associated with risk of developing asthma in people over 60 years of age based on XGBoost modeling

**Fig. 2:**
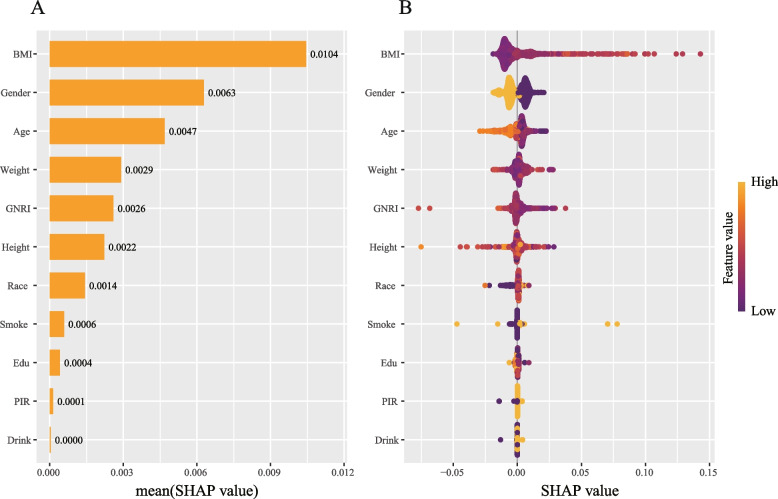
Explanation of the machine learning model using SHAP summary and dependency plots. **A**-**B** The importance matrix and SHAP summary plot illustrate the contributions of all variables to the XGBoost model

The XGBoost model was used to evaluate the relative contribution of multivariate variables to the risk of asthma in people aged > 60 years, and the model was parsed in detail using the Shapley Additive Explanations (SHAP) method. The mean squared error (MSE) of the XGBoost model was 0.08, indicating a minimal squared deviation between the predicted and actual values of the target variable. Furthermore, the root MSE was 0.28, which further validates the high accuracy of the model predictions. The order of importance of the variables revealed that BMI was the most important predictor of asthma in people aged > 60 years (mean SHAP value of 0.0104), significantly higher than that of the other variables. Gender (0.0063), age (0.0047), and weight (0.0029) followed closely. The model also emphasized the significance of GNRI, a new nutritional risk indicator strongly associated with the risk of developing asthma in individuals aged > 60 years (Fig. 2A). The SHAP dependency plot further elucidated the specific contributions of each variable to the model predictions (Fig. 2B). The findings revealed a significant positive correlation between the contribution of BMI to the risk of developing asthma in individuals aged > 60 years, with a significant increase in SHAP values as BMI increased, indicating a significant increase in asthma in individuals aged > 60 years. GNRI exhibited a nonlinear relationship with asthma in individuals aged > 60 years, with high GNRI values (low nutritional risk) contributing significantly more to the asthma. Height, race, smoking, and education exhibited relatively minor effects, but the distribution of their SHAP values suggested that certain subgroups (such as specific races or levels of education) may have a higher risk of developing asthma. Although the mean SHAP values of other variables, such as poverty income ratio (PIR) (0.0001) and alcohol consumption (0.0000), were lower, they were still predictive of asthma.

### Relationship between asthma and GNRI in elderly individuals aged 60 and above, and its subgroups, based on RCS curves

**Fig. 3:**
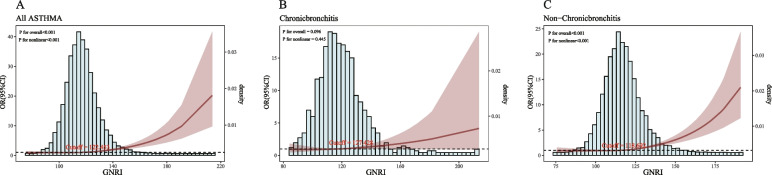
RCS curves for the relationship between GNRI and asthma in elderly individuals aged 60 and above. Note: Covariates included race, education, emphysema, and chronic bronchitis diagnoses

The relationship between GNRI and asthma in individuals aged 60 and above was examined using RCS curves, adjusting for all relevant covariates. A significant linear trend was observed between the GNRI and asthma in the elderly (*P* for overall trend < 0.001), and a significant nonlinear relationship was observed (*P* for nonlinearity < 0.001). The curve demonstrated that the risk of asthma in elderly individuals was significantly increased when the GNRI reached or exceeded 122.453, with the 95% CI gradually widening (Fig. 3A). Furthermore, in the chronic bronchitis subgroup (Fig. 3B), the relationship between the GNRI and asthma in individuals aged 60 and above was non-significant (*P* = 0.096), and no nonlinear trend was observed (*P* for nonlinearity = 0.445). However, a significant linear relationship (*P* < 0.001) and a pronounced nonlinear trend (*P* for nonlinearity < 0.001) were observed in the non-chronic bronchitis subgroup. The risk of asthma in the elderly significantly increased when the GNRI reached 118.692 (Fig. 3C).

### Relationship between GNRI and baseline characteristics subgroups

**Fig. 4:**
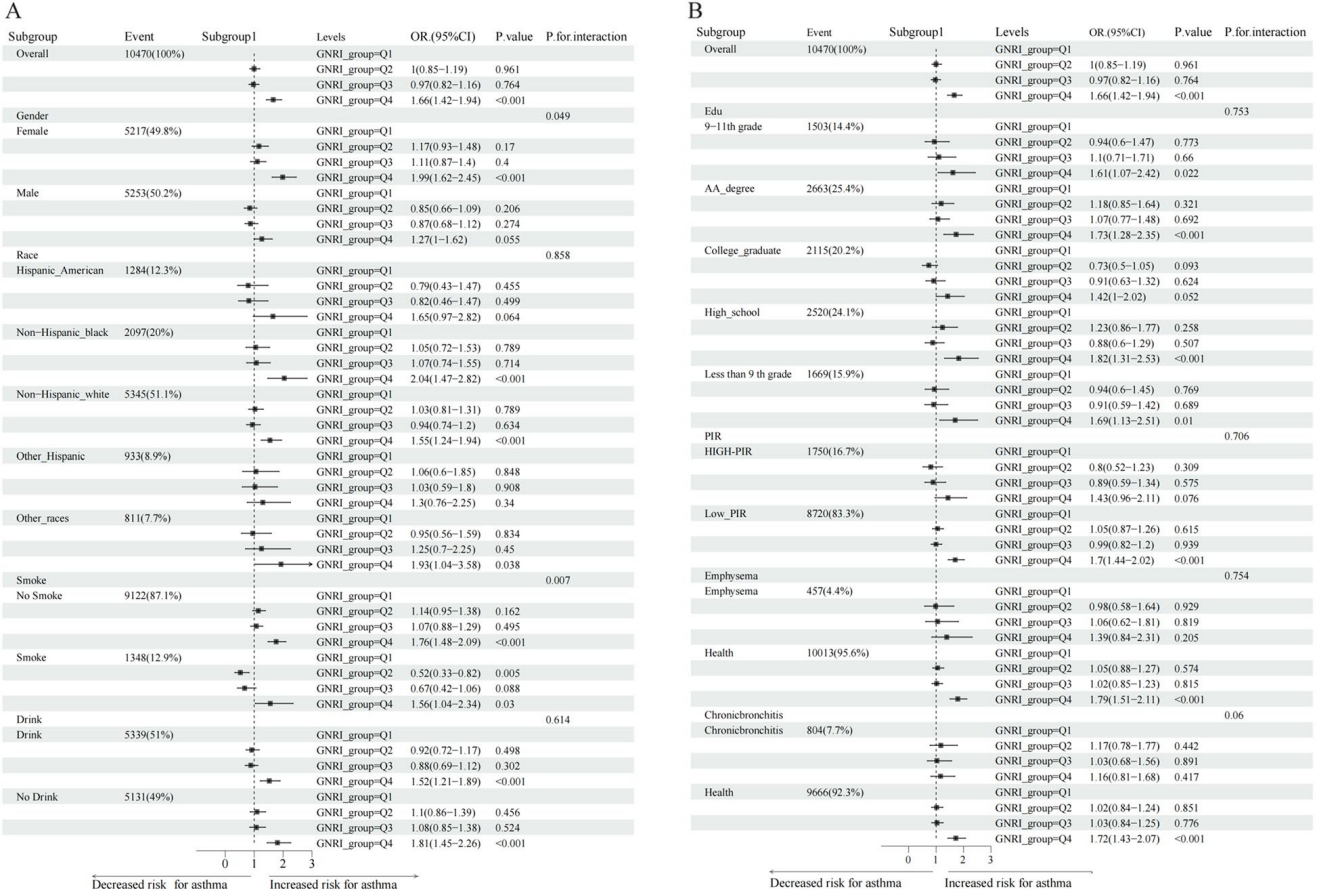
Relationship between GNRI and asthma in elderly individuals aged 60 and above by baseline characteristic subgroups (**A**-**B**). Note: 9th–11th Grade: 9th–11th Grade, AA Degree: Associate degree, College Graduate: College graduate, Less Than 9th Grade: Less than 9th grade; PIR ≤ 4.99: Low, PIR ≥ 5: High; Covariates: race, education, emphysema diagnosis, and chronic bronchitis diagnosis

This study used multivariable logistic regression to analyze the association between GNRI and asthma in elderly individuals aged 60 and above, with stratified analysis in different baseline characteristic subgroups (Fig. 4). The findings indicated that the highest GNRI group (Q4, highest 25%) was significantly associated with an increased risk of asthma in elderly individuals (OR = 1.66, 95% CI: 1.42–1.94, *P* < 0.001) compared to the lowest GNRI group (Q1, lowest 25%). The risk in Q3 was also elevated but to a lesser extent (OR = 0.97, 95% CI: 0.82–1.16, *P* = 0.764).

GNRI did not have a significant effect on the male subgroup. However, the higher GNRI groups in females exhibited a significantly higher risk of asthma (Q4 versus Q1, OR = 1.99, 95% CI: 1.62–2.45, *P* < 0.001), implying a more robust predictive role of GNRI for asthma in older women.

The highest GNRI group (Q4) was significantly associated with an increased asthma among non-Hispanic White and non-Hispanic Black individuals (Q4 versus Q1, OR = 1.55, 95% CI: 1.24–1.94, *P* < 0.001 and Q4 versus Q1, OR = 2.04, 95% CI: 1.47–2.82, *P* < 0.001, respectively). Conversely, no significant associations were observed among the other racial subgroups.

The association between GNRI and asthma was significant in non-smokers, with the highest GNRI group (Q4) significantly increasing the asthma (OR = 1.76, 95% CI: 1.48–2.09, *P* < 0.001). However, no significant association was observed in the smoker subgroup. Similarly, GNRI was significantly associated with an increased asthma among drinkers (Q4 versus Q1, OR = 1.52, 95% CI: 1.21–1.89, *P* < 0.001), and the same pattern was observed in non-drinkers (Q4 versus Q1, OR = 1.81, 95% CI: 1.45–2.26, *P* < 0.001) (Fig. 4A).

For individuals with varying levels of education (9th–11th Grade, AA Degree, College Graduate, < 9th Grade), compared to Q1, the highest GNRI group (Q4) was significantly associated with higher asthma in the following groups: some college (OR = 1.73, 95% CI: 1.28–2.35, *P* < 0.001), high school (OR = 1.82, 95% CI: 1.31–2.53, *P* < 0.001), and < 9th Grade (OR = 1.69, 95% CI: 1.13–2.51, *P* = 0.01). However, no significant associations were observed in the other educational subgroups.

In the PIR ≤ 4.99 group (low income), the association between GNRI and asthma was more pronounced, with the highest GNRI group (Q4) significantly increasing the asthma (OR = 1.70, 95% CI: 1.44–2.02, *P* < 0.001). No significant association was observed in the PIR > 5 (high-income) group.

GNRI was significantly associated with asthma in individuals without emphysema, with the highest GNRI group (Q4) significantly increasing the risk of asthma (OR = 1.79, 95% CI: 1.51–2.11, *P* < 0.001). However, no significant association was observed in patients with emphysema.

### Exploring potential mechanisms of association

Furthermore, using a fully adjusted model, this study evaluated the relationship between GNRI and inflammatory markers. The results demonstrated a statistically significant negative correlation between GNRI and Globulin (β = –0.014, 95% CI: –0.021 to –0.007, *P* < 0.001; Table 3). Similarly, significant positive correlations were observed between GNRI and the following markers: Eo count (β = 0.216, 95% CI: 0.037–0.402, *P* = 0.018), Ba count (β = 0.103, 95% CI: 0.046–0.161, *P* < 0.001), WBC count (β = 0.029, 95% CI: 0.016–0.044, *P* < 0.001), RBC count (β = 0.693, 95% CI: 0.624–0.762, *P* < 0.001), Neutrophil count (β = 0.059, 95% CI: 0.038–0.081, *P* < 0.001), Alb (β = 0.133, 95% CI: 0.122–0.144, *P* < 0.001), and Monocyte count (β = 0.267, 95% CI: 0.117–0.418, *P* < 0.001). We assessed the correlation between the GNRI and inflammatory markers in the control group to further eliminate bias in asthma in the general population (Table 3).

**Table 3:** The association between GNRI and asthma-related Indicators in overall population and controls

Mediating factors	Overall (*N* = 10,470)		Asthmatic patients (*N* = 1352)		Non-asthmatic patients (*N* = 9118)	
	β value (95% CI)	*P* value	β value (95% CI)	P value	β value (95% CI)	*P* value
Eo count	0.216 (0.037, 0.402)	0.018	–0.477 (–0.944, –0.015)	0.043	0.289 (0.089, 0.498)	0.004
Ba count	0.103 (0.046, 0.161)	< 0.001	0.094 (–0.062, 0.253)	0.24	0.099 (0.039, 0.162)	0.001
WBC count	0.029 (0.016, 0.044)	< 0.001	0.067 (0.026, 0.111)	0.001	0.021 (0.008, 0.036)	< 0.001
RBC count	0.693 (0.624, 0.762)	< 0.001	0.574 (0.382, 0.767)	< 0.001	0.732 (0.658, 0.807)	< 0.001
Neutrophil count	0.059 (0.038, 0.081)	< 0.001	0.096 (0.039, 0.154)	0.001	0.047 (0.025, 0.070)	< 0.001
Alb	0.133 (0.122, 0.144)	< 0.001	0.082 (0.052, 0.112)	< 0.001	0.145 (0.133,0.157)	< 0.001
Globulin	–0.014 (–0.021, –0.007)	< 0.001	–0.001 (–0.02, 0.017)	0.891	–0.017 (–0.025, –0.010)	< 0.001
Monocyte count	0.267 (0.117, 0.418)	< 0.001	–0.077 (–0.514, 0.361)	0.730	0.312 (0.152, 0.478)	< 0.001

*Abbreviations Eo count* Eosinophilic Granulocyte count, *Ba count* Basophilic granulocyte count, *WBC count* White blood cell count, *RBC count* Red blood cell count, *Neutrophil count* Alb, Albumin, *Globulin* Monocyte count

### Sensitivity analysis

A series of sensitivity analyses were performed to evaluate the robustness of the main findings of this study. First, the results remained consistent when individuals with other comorbidities were excluded from the control group (Table S1). Further subgroup analysis revealed consistent associations between the GNRI and asthma in elderly individuals aged 60 and above (Fig. 3A).

Following propensity score matching (PSM), the study included 2,704 participants, of whom 1,352 were elderly individuals with asthma, and 1,352 were without asthma (Table S2). GNRI remained significantly associated with increased asthma in this subgroup, irrespective of the adjusted and unadjusted models (Table S1, Model 1: OR = 1.016, 95% CI: 1.01–1.022, *P* < 0.001; Model 2: OR = 1.017, 95% CI: 1.011–1.023, *P* < 0.001). The adjusted models also confirmed the association between GNRI and asthma in the PSM cohort. As GNRI increased, the risk of asthma in elderly individuals aged 60 and above progressively increased (Model 2, Q4 versus Q1, OR = 1.017, 95% CI: 1.011–1.023, *P* < 0.001, *P* for trend < 0.001; Table S2).

## Discussion

GNRI is a valuable tool for evaluating the nutritional status of elderly individuals and is frequently used in clinical and research settings. Malnutrition, particularly indicated by reduced GNRI values, is a common problem among the elderly and is closely linked to the development and worsening of various chronic diseases. In this retrospective cohort study, we assessed the nutritional status of 1,352 elderly individuals with asthma using the GNRI. The risk regression models revealed that the GNRI positively correlated with asthma in individuals aged > 60 years. Furthermore, restricted cubic spline regression models identified a significant association between GNRI and asthma. Subgroup analysis further confirmed that GNRI was associated with asthma in elderly individuals, indicating that malnutrition may be associated with asthma and could potentially be modifiable. This study underscores that asthma is more prevalent among elderly individuals with a low GNRI, particularly those who are malnourished.

These findings highlight the significance of prioritizing nutritional status for respiratory health in the elderly population. Nutritional status is important for predicting the risk of various diseases. As an effective marker of malnutrition, lower GNRI values indicate that elderly individuals may experience inadequate or impaired nutrient absorption, leading to immune system dysfunction and increased susceptibility to diseases [19]. Furthermore, low GNRI levels may be linked to common chronic diseases in the elderly, which in turn affect respiratory function and increase the risk of asthma [20]. GNRI can be calculated without patient participation or guidance from nutrition specialists, highlighting its practical value [21]. Currently, research on the relationship between GNRI and asthma is limited. However, our study suggests that GNRI may be an important predictor of asthma in the elderly. GNRI is primarily calculated based on weight, height, and serum Alb levels, all closely related to asthma. As a result, GNRI not only reflects the nutritional status of the elderly but also evaluates key health factors related to asthma, rendering it a more effective indicator for assessing asthma in this population.

Inflammation is closely linked to the onset of asthma, and nutritional status significantly influences inflammatory responses. The immune system of elderly individuals is often in a state of chronic subclinical inflammation, rendering them more susceptible to asthma and chronic obstructive pulmonary disease (COPD). Chronic low-grade inflammation may alter the immune response and function of the airways, leading to the onset and exacerbation of asthma [22, 23]. Besides, GNRI may be associated with asthma as a nutritional status assessment tool. The inflammatory characteristics of elderly patients with asthma typically involve neutrophils and monocytes rather than eosinophils, implying that the inflammatory profile may evolve with age. This may explain the differences in the clinical manifestations of asthma between older and younger patients.

Increasing evidence suggests that malnutrition and a low GNRI are closely associated with chronic low-grade inflammation, a core mechanism in the development of asthma [24]. Asthma, a common chronic inflammatory airway disease, has a complex and variable pathogenesis, with airway remodeling playing a key role in its chronic progression [25]. Airway remodeling, characterized by chronic inflammation, may cause pathological changes such as epithelial fragility, goblet cell hyperplasia, increased submucosal mucous glands, angiogenesis, increased deposition of airway wall matrix, increased airway smooth muscle mass, thickening of the airway wall, and abnormal elastic fibers. These changes significantly elevate airway resistance and hyperresponsiveness, important pathological features of refractory asthma [26]. Airway inflammation, a core pathological feature of asthma, is closely associated with airway remodeling [27]. Chronic inflammation leads to the release of Th2 cytokines, such as interleukin-4 (IL-4), IL-5, and IL-13, from the airway epithelium, promoting subepithelial fibrosis, smooth muscle cell proliferation, eosinophil activation, and infiltration. Simultaneously, fibroblasts and smooth muscle cells are activated, exacerbating their proliferation and activation [28]. The primary manifestation of immune dysregulation in the pathological state of asthma is the abnormal activation of the Th2 immune response. Cytokines released by Th2 cells, such as IL-4, IL-5, and IL-13, promote the proliferation and activation of eosinophils, mast cells, and goblet cells in the airways, triggering and exacerbating airway inflammation [29]. Numerous inflammatory cells, such as eosinophils, mast cells, lymphocytes, and neutrophils, infiltrate the airway walls. These cells release IL, prostaglandins, and TGF-β, which directly damage airway tissues and activate structural cells, such as fibroblasts and smooth muscle cells, indirectly promoting airway remodeling [30–32]. Moreover, Th17 cells secrete cytokines, including IL-17 and IL-22, which can induce the infiltration and activation of neutrophils and other inflammatory cells in the airways, further exacerbating inflammation and the remodeling process [33]. Furthermore, studies have demonstrated that oxidative stress results in airway remodeling, and malnutrition may contribute to oxidative stress, negatively affecting tissues [34]. Therefore, our study further analyzed the correlation between GNRI and various inflammatory markers (such as Eo count, WBC count, and neutrophil count) and found significant positive correlations, implying that malnutrition may be associated with chronic low-grade inflammation, which is linked to asthma.

GNRI is closely related to Alb. As a direct marker of protein nutritional status, low Alb levels are often associated with malnutrition, reduced immune function, and an increased risk of chronic disease [35]. Low Alb and BMI indicate malnutrition and are essential components of GNRI [16]. Studies have demonstrated that chronic inflammation plays a role in lowering Alb levels, and a low BMI may be a distinct indicator of malnutrition [36]. This is consistent with our findings. The combination of inflammation and low nutritional status may further exacerbate the risk of asthma in the elderly population. Our findings demonstrated a significant relationship between GNRI and Alb, implying that low nutritional status is associated with inflammatory responses. Consequently, low Alb levels in the elderly may indicate suppressed immune function, rendering them more susceptible to respiratory infections and an increased risk of asthma. The combined effect of inflammation and low nutritional status may further exacerbate the risk of asthma. Improving nutritional status, particularly enhancing protein and micronutrient intake, may help reduce inflammation and lower the risk of asthma in the elderly. Further analysis indicated that low Alb levels may directly affect the function of airway immune cells, such as inhibiting eosinophil activity and migration. This may explain why asthma symptoms in elderly patients differ from those in younger individuals. The immune response in the elderly is relatively delayed, particularly in chronic diseases, with more active neutrophils and monocytes. Consequently, nutritional supplementation, particularly with proteins and immune boosters, may reduce asthma and other immune-related diseases in the elderly.

This study revealed that elderly individuals with low GNRI levels are at a higher risk of asthma, consistent with previous studies. However, there are some differences. Our findings are consistent with those of Kiesswetter et al., who indicated that malnutrition is associated with an increased risk of respiratory diseases in the elderly [37]. They revealed that a low GNRI is related to a decline in lung function and may also lead to increased chronic inflammation, which could be one of the key mechanisms underlying the increased risk of asthma. Furthermore, our study provides evidence of the association between asthma and GNRI in the elderly, implying that a low GNRI may predispose elderly individuals to asthma due to immune dysfunction, enhanced inflammation, and airway structural changes. Moreover, other studies have explored the role of GNRI in patients with COPD and identified that a low GNRI is related to COPD. However, its role in patients with asthma has not been thoroughly investigated [38]. Our research fills this gap, indicating that poor nutritional status may increase the risk of asthma in the elderly, in addition to its association with COPD. Conversely, their research focused more on how malnutrition in patients with COPD influences disease progression, whereas our study emphasizes GNRI as a potential marker for predicting asthma.

In conclusion, our study highlights the role of GNRI in predicting asthma and explores its underlying mechanisms, including chronic inflammation, immune dysfunction, and lung damage. However, further prospective studies are required to validate the efficacy of GNRI as a predictive marker for geriatric asthma and to explore the role of various demographic factors, such as age, gender, and other influencing variables, in this relationship.

### Strengths and limitations

In this study, we used a retrospective cohort design to investigate the correlation between the GNRI and asthma in the elderly. These findings are consistent with those of previous studies, indicating that low GNRI levels are associated with an increased risk of respiratory diseases in the elderly. The major advantage of this design is its ability to analyze existing large-scale medical data, which increases the feasibility of the study and allows for a large sample size in a relatively short time. Furthermore, retrospective studies are suitable for analyzing long-term health changes. They can help explore the role of the GNRI as a nutritional assessment tool for predicting the risk of asthma.

Several important limitations should be acknowledged. First, as an observational study, our design precludes causal inference and may be susceptible to reverse causation. Specifically, retrospective cohort studies like ours may also be prone to Berkson's or Neyman's bias, which commonly occurs in studies based on inpatient or medical data. This bias can result in the overrepresentation of patients with longer or more severe disease courses, while neglecting those with shorter or milder cases, potentially influencing the true association between GNRI and asthma. Furthermore, the study may be limited by insufficient control of confounding factors and the lack of dynamic monitoring of changes in nutritional status, as the data were retrospectively collected. Therefore, the causal relationship between GNRI and asthma and other potential mechanisms should be further investigated in future studies to provide scientific evidence for the prevention and control of asthma in the elderly.

## Conclusions

This study demonstrates that GNRI levels are significantly associated with asthma in the elderly. Such studies would play a crucial role in enhancing early risk identification, improving stratification strategies, and promoting cost-effective screening approaches, particularly in clinical practice and large-scale epidemiological settings.

## Supplementary Information

Supplementary Material 1: Table S1. Relationship between GNRI and the prevalence of asthma after PSM). Table S2. The relationship between GNRI and the prevalence of asthma further excludes patients with tumors from the control group).

## Data Availability

Ethics approval was obtained from the NCHS Ethics Review Committee, and participants provided written informed consent. The NHANES was authorized by the National Center for Health Statistics (NCHS) Ethics Review Committee, and all participants completed written informed consent forms before participation. Publicly available datasets are available online for this study. The repository/repositories name and accession numbers are available online at http://www.cdc.gov/nchs/nhanes.htm (accessed on 31 Dec 2024). Since the secondary analysis did not need further institutional review board approval, the ethical review and approval were waived.

## Abbreviations

GNRI: Geriatric Nutritional Risk Index
OR: the odds ratio
Eo count: eosinophils
Ba count: basophils
WBC count: white blood cells
RBC count: red blood cells
Neutrophil count: neutrophils
Alb: albumin
Monocyte count: monocytes
PRD: Protein-restricted diet
RCTs: Randomized controlled trials
OVA: Ovalbumin
NCHS: the National Center for Health Statistics
CDC: the Centers for Disease Control and Prevention
MEC: the Mobile Examination Centers
MCQ010: he Medical Condition Questionnaire
Alb: albumin
Globulin: globulin
DEMO: determined from the demographic file
RCS: Restricted Cubic Splines
CIs: Confidence intervals
IQR: Interquartile ranges
PSM: Propensity score matching
ASM: Airway smooth muscle
COPD: Chronic obstructive pulmonary disease
