# Supplementary material for: Association between geriatric nutritional risk index (GNRI) and asthma in elderly individuals aged 60 and above: a cross-sectional study of the NHANES 2005–2018
Source: BMC Pulm Med. 2025 Sep 24;25:426. doi: 10.1186/s12890-025-03830-7 (PMC12462012; doi:10.1186/s12890-025-03830-7)
Supplement: Supplementary file 1 — Supplementary Material 1: Table S1. Relationship between GNRI and the prevalence of asthma after PSM). Table S2. The relationship between GNRI and the prevalence of asthma further excludes patients with tumors from the control group). [file 12890_2025_3830_MOESM1_ESM.docx]

Table S1. Relationship between GNRI and the prevalence of asthma after PSM (multifactor regression OR (95% CI)).

| Variable | Model 1 | Model 2 |
| --- | --- | --- |
| **GNRI** | 1.016(1.01,1.022) | 1.017(1.011,1.023) |
| *P*-value | *P* < 0.001 | *P* < 0.001 |
| **GNRI, quartile** |  |  |
| Q1 (< 109.61) | Ref | Ref |
| Q2 (109.61–116.71) | 1.061 (0.857, 1.314) | 1.074 (0.866, 1.333) |
| Q3 (116.71–125.48) | 1.181 (0.954, 1.462) | 1.214 (0.978, 1.508) |
| Q4 (≥ 125.48) | 1.629 (1.315, 2.021) | 1.679 (1.35, 2.092) |
| *P*-trend | *P* < 0.001 | *P* < 0.001 |

Model 1 was unadjusted.

Model 2 was adjusted for race, education, and emphysema.

GNRI, Geriatric nutritional risk index; PSM, propensity score matching.

Table S2. The relationship between GNRI and the prevalence of asthma further excludes patients with tumors from the control group. (multifactor regression OR (95% CI)).

| Variable | Model 1 | Model 2 |
| --- | --- | --- |
| **GNRI** | 1.016 (1.01,1.022) | 1.017 (1.011,1.023) |
| *P*-value | *P* < 0.001 | *P* < 0.001 |
| **GNRI, quartile** |  |  |
| Q1 (< 109.61) | Ref | Ref |
| Q2 (109.61–116.71) | 1.061 (0.857, 1.314) | 1.074 (0.866, 1.333) |
| Q3 (116.71–125.48) | 1.181 (0.954, 1.462) | 1.214 (0.978, 1.508) |
| Q4 (≥ 125.48) | 1.629 (1.315, 2.021) | 1.679 (1.35, 2.092) |
| *P*-trend | *P* < 0.001 | *P* < 0.001 |

Model 1 was unadjusted.

Model 2 was adjusted for race, education, and emphysema.

GNRI, Geriatric nutritional risk index; PSM, propensity score matching.
